# Clinical and radiological outcome of arthrocentesis followed by autologous blood injection for treatment of chronic recurrent temporomandibular joint dislocation

**DOI:** 10.4317/jced.53812

**Published:** 2017-08-01

**Authors:** Jinesh Patel, Kumar Nilesh, M. I Parkar, Alpesh Vaghasiya

**Affiliations:** 1 MDS (OMFS), Post graduate student, Department of Oral and Maxillofacial Surgery, School of dental scienes,KIMSDU, Karad; 2 MDS (OMFS), Reader, Department of Oral and Maxillofacial Surgery, School of dental scienes,KIMSDU, Karad; 3 MDS (OMFS), Professor & HOD, Department of Oral and Maxillofacial Surgery, School of dental scienes,KIMSDU, Karad

## Abstract

**Background:**

This study was conducted to evaluate the functional outcome and MRI findings of arthrocentsis followed by autologous blood injection (ABI) into the joint space for management of chronic recurrent TMJ dislocation.

**Material and Methods:**

Total ten patients with bilateral chronic recurrent condylar dislocation were included in the study. Arthrocentesis of both TMJ was performed on each patient, followed by the injection of 2 ml of autologous blood into the superior joint compartment and 1 ml onto the outer surface of the joint capsule. Preoperative and postoperative assessment included; thorough history, clinical examination of TMJ, maximal mouth opening, frequency of dislocation, TMJ radiographs (open and closed mouth position), MRI, recurrence and presence of facial nerve paralysis.

**Results:**

At the end of 3 months follow-up 8 patients (80%) had successful outcome with no further episodes of dislocation, whereas two patients reported with recurrence. Post-operative MRI showed significant improvement after ABI, compared to pre-operative MRI. There were no degenerative changes to the bony and soft tissue components of TMJ.

**Conclusions:**

ABI is a simple, safe, minimally invasive and cost-effective technique for treatment of chronic recurrent TMJ dislocation. MRI evaluation showed an improvement in the anatomical and spatial relationship of the osseous and soft tissue components of the TMJ.

** Key words:**TMJ lavage, luxation, fibrosis, magnetic resonance imaging.

## Introduction

Dislocation of the Temporomandibular Joint (TMJ) is a pathophysiologic condition that is challenging to manage in clinical practice. In TMJ dislocation, the condyle reaches a position in front of the articular eminence, on wide mouth opening. It can be caused by abnormalities in the osseous architecture of the joint, laxity of TMJ ligament or due to reduced muscle tension (1). It is broadly classified into the three types; acute, chronic, and chronic recurrent (2). In chronic recurrent dislocations an individual experiences multiple episodes of dislocations as a result of everyday activities like excessive mouth opening, laughing and yawning. It can become physically and emotionally distressing, thereby compromising quality of life. Untreated and progressive disease may cause injury to the disk, capsule, and ligaments, leading to progressive internal derangement of TMJ and arthritic joint degeneration (3).

Many surgical and non-surgical techniques for treatment of chronic recurrent TMJ dislocation are described in the literature. Aim of surgical treatment is to restrict the condylar movement beyond the articular eminence, by creating a mechanical obstruction along the condylar pathway (4). Surgical interventions include capsular plication, reduction or augmentation of the articular eminence, temporalis tendon scarification, lateral pterygoid myotomy, and condylectomy (5). These procedures require hospitalization, general anesthesia and surgical access to TMJ area. Treating TMJ by surgical intervention require careful surgical dissection, due to complex anatomy of TMJ. It carries risk of complications such as facial nerve injury, altered sensation, swelling, pain and infection. Conservative method to treat chronic recurrent TMJ dislocation has become popular over the past few decades. Various treatment options for conservative management include; injection of botulinum toxin in the muscles of mastication, use of sclerosing agents (alcohol, sodium tetradecyl sulfate, sodium psylliate and morrhuate sodium) and immobilization of jaw with arch bars and ligature wires (intermaxillary fixation) (6-8).

Autologous blood injection (ABI) for treatment of TMJ dislocation was first reported by Brachmann in 1964 (9). Although subsequently there were successful reports of this treatment modality, its use in clinical practice did not gain popularity due to some unclear reason. Hasson and Nahlieli reintroduced this treatment modality in 2001 (1). Oshiro N *et al.* in 2014, studied the clinical and MRI findings following ABI for treatment of habitual TMJ dislocation (10). They found ABI to be minimally invasive, effective and safe treatment. The principle behind ABI is to restrict mandibular movements by inducing fibrosis in upper joint space, pericapsular tissues or both by injecting blood into TMJ. In this conservative method patient’s own blood is injected in TMJ, thereby avoiding any chance of allergic reaction and postoperative infection.

In the present study clinical and radiolographic evaluation was carried to assess the outcome of ABI for management of patients with chronic recurrent TMJ dislocation. Quantifiable parameters, including linear and angular measurements were carried on pre and post injection MRI to standardise the treatment outcome.

## Material and Methods

This study was approved by the krishna institute of medical sciences deemed university institutional review board and all participants signed an informed consent agreement. Ten patients with bilateral chronic recurrent TMJ dislocation were selected for the study. The preoperative assessment included; detailed case history, clinical TMJ examination, extraoral TMJ radiograph and MRI (closed and open mouth position). Patients with minimum two episodes of TMJ dislocation in the immediate past 6 months and with radiological evidence of displacement of condylar head beyond the articular eminence on wide mouth opening (on plane radiograph and MRI) were selected for ABI.

-Clinical procedure of arthrocentesis & ABI:

The preauricular area was prepared with antiseptic solution and isolated with sterile drapes. Auriculotemporal nerve block was given on both sides using 2% lidocaine with 1:100,000 epinephrine. A straight line was drawn from the middle of the tragus to the lateral canthus. The Point A was located along the canthotragal line, 10 mm from the middle of the tragus and 2 mm below the line. The Point B was placed 10 mm further along the line and 10 mm below it. An 18-gauge needle was inserted at Point A, for entry of lavage solution and second 18-guage needle was inserted at point B to allow for fluid to exit during the lavage. The joint was slowly flushed with approximately 10 mL of lactated Ringers solution. Following the arthrocenthesis procedure needle at point B was removed. Three millilitres of blood was withdrawn from the patient’s anticubital fossa. Two millilitres of blood was injected into the Superior joint compartment (SJC) through needle at Point A and remaining one millilitre was injected around the capsule. This procedure was repeated on the opposite side in the same manner.

Oral antibiotic (amoxicillin clavulanate 625mg; 12 hourly) and analgesic (diclofenac 50 mg + paracetamol 500mg) was prescribed for 5 days to every patient. During the first week, patients were advised to restrict mouth opening to less than 20 mm, and follow a diet limited to soft food only. Starting at 2 weeks, the patients were given jaw rehabilitation exercises with gradual and controlled range of motion in front of a mirror. They were advised to advance their diet as tolerated.

-Parameters evaluated:

A) Clinical Parameters 

1) Pain: It was evaluated (for presence or absence) during opening and closing of mouth, preoperatively and post operatively after 1 week, 4 weeks and 3 months.

2) Maximum inter-incisal opening (MIO): It was measured (in mm) from incisal edge of 11 to 41, in maximum mouth open position, pre-operatively and at 3 months post-operative period.

3) Frequency of TMJ dislocation: It was assessed pre-operatively, 4 weeks and 3 months post-operative period.

4) Facial nerve injury: It was evaluated at 1 week, 4 weeks and 3 months post operative period.

5) Clicking sound: Presence of clicking sounds during mouth opening and closing was rated by operator as follows; 0 (absent), 1 (low), 2 (mild), 3 (intense) or 4 (severe). It was evaluated pre-operatively and at 4 weeks and 3 months post operative period.

B) Radiological evaluation: Two dimensional TMJ view in maximum open and closed mouth position was taken at pre-operative and 3 months post-operative period after ABI.

C) Parameters on MRI

MRI of TMJ was done pre-operatively and at 3 months post-operative period. The parameters studied included.

Parameter 1: angle between condylar head and TMJ disk

In the sagital view of TMJ in maximum open mouth position, an imaginary circle was drawn with posterior border of condylar head as the outer circumference of the circle. A line was drawn through twelve o’ clock position, from centre of circle. A second line was drawn from centre of the circle, passing through junction of the posterior band and the bilaminar zone of TMJ disk. The angle formed by intersection of these two lines was measured.

Parameter 2: distance between condyle and external auditory canal (EAC)

The distance from the posterior most point of the condyle head and the anterior most point of the EAC was measured in millimetres in pre operative and post operative MRI of TMJ (open mouth position).

Parameter 3: type of the condyle position

The position of mandibular condyle head in relation to articular eminence was evaluated in maximum mouth open position. A horizontal line parallel to frankfurt plane was drawn from the lowest point of the curvature in the articular eminence. A vertical line was drawn passing from the centre of the articular eminence and crossing this horizontal line. Seven types of condyle relations were defined: type 1 (posterior-inferior), type 2 (posterior-superior), type 3 (posterior and at the same level), type 4 (inferior), type 5 (anterior-inferior), type 6 (anterior-superior) and type 7 (anterior and at the same level).

## Results

Out of 10 patients participating in this study, six were male and four were female; having mean age of 28.9 years (ranging from 21 to 55 years) ( Table 1). All of the patients tolerated the procedure well without any complications. There were no incidences of facial nerve palsy, deviation in mouth opening or unusual inflammatory reaction after ABI.

**Table 1:**
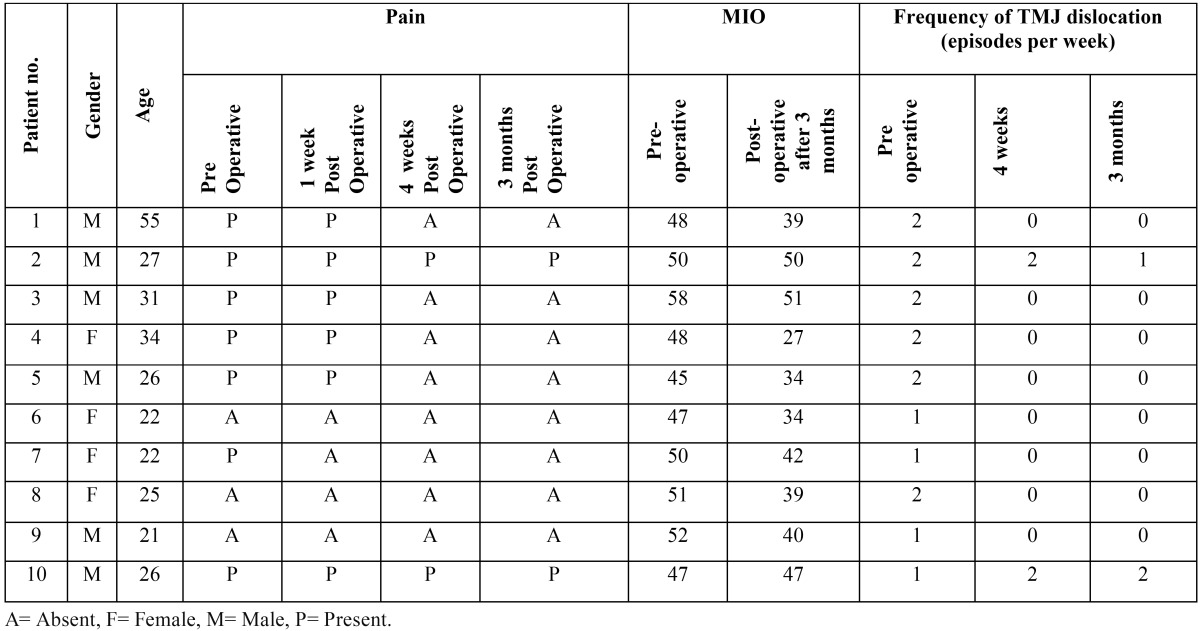
Distribution of age, sex, pain, MIO and frequency of TMJ dislocation, before and after ABI.

Pre-operatively pain was present in 7 (70%) patients and absent in 3 (30%) patient. Post operatively at 1 week, pain was present in 6 (60%) patients, at 4 weeks pain was present in 2 (20%) patients and at 3 months pain was present in 2 (20%) patients ( Table 1).

Pre-operative MIO ranged from 45 to 58 mm with an average of 49.6 mm. At three months post-operative period MIO ranged from 27 to 51 mm with an average of 40.30 mm. The reduction of MIO between pre-operative and post-operative period ranged from 0 to 21 mm, with an average reduction of 9.30 ± 6.21 mm ( Table 1).

Frequency of TMJ dislocation measured pre-operatively, was one episode per week in 4 (40%) patients and 2 episodes per week in 6 (60%) patients. At four weeks follow-up 3 patients presented with recurrence. Subsequently at 3 months follow-up, 2 patients presented with 1 -2 episodes of dislocation per week, whereas 8 patients had no episode of TMJ dislocation ( Table 1).

Clicking sound evaluated pre-operatively, showed no sound present in 1 (10%) patient, mild sound present in 3 (30%) patients, intense sound present in 5 (50%) patients and severe sound present in 1 (10%) patient. Post-operatively (at 4 weeks), no sound was present in 5 (50%) patients, mild sound was present in 3 (30%) patients, intense sound was present in 1 (10%) patient and severe sound was present in 1 (10%) patient. At 3 months follow-up, no sound was present in 6 (60%) patients, mild sound was present in 2 (20%) patients, intense sound was present in 1 (10%) patient and severe sound was present in 1 (10%) patient ( Table 2).

**Table 2:**
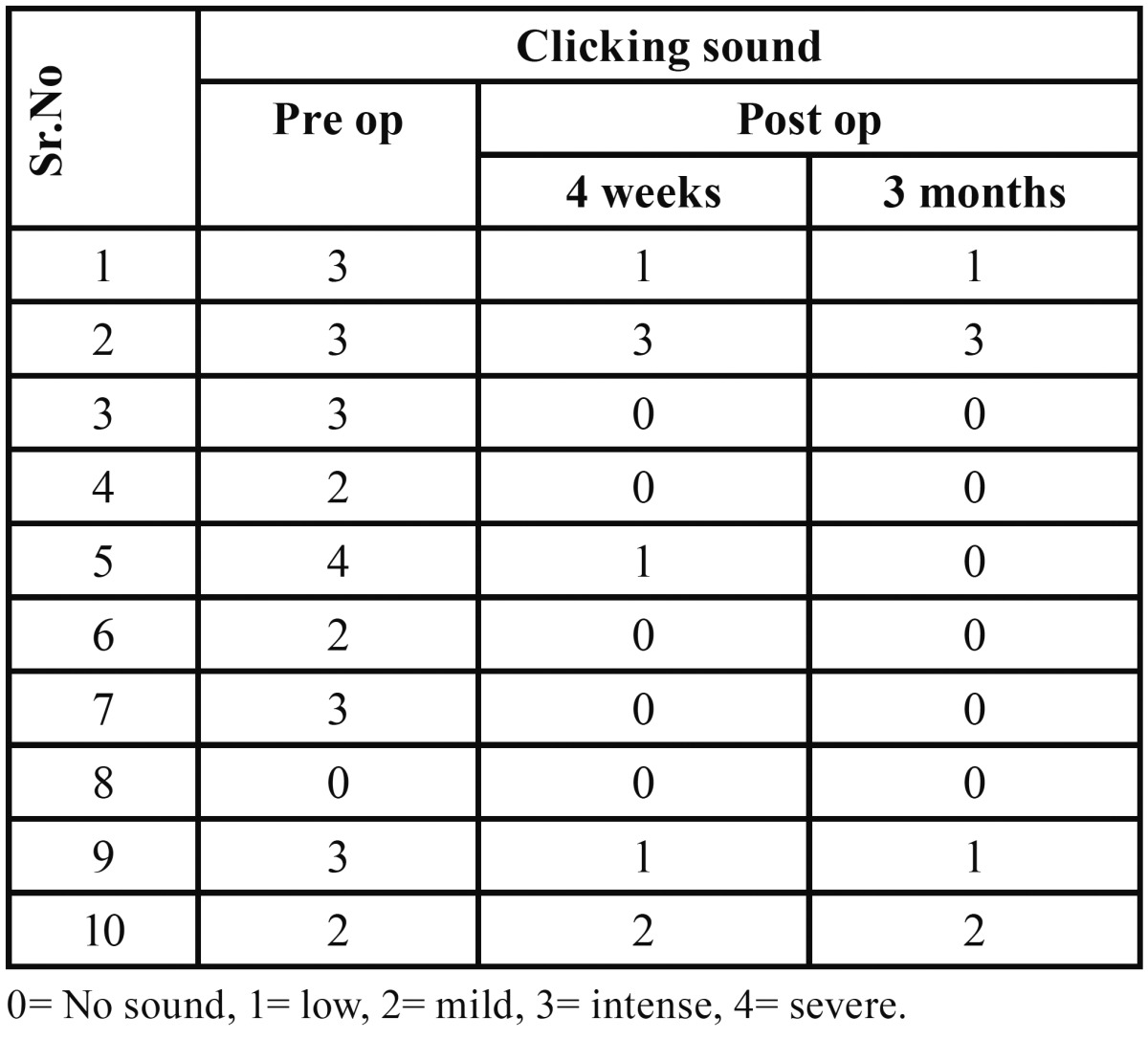
Clicking sound pre-operatively and at 4 weeks and 3 months post-operative period.

Evaluation of the post-operative TMJ views at maximum mouth open position taken 3 months after ABI therapy showed condyle to be at the apex of articluar eminence in 8 patients, indicating reduction in the abnormal anterior condylar movement. Two patients did not show any significant reduction of the anterior condylar position.

**Figure 1:**
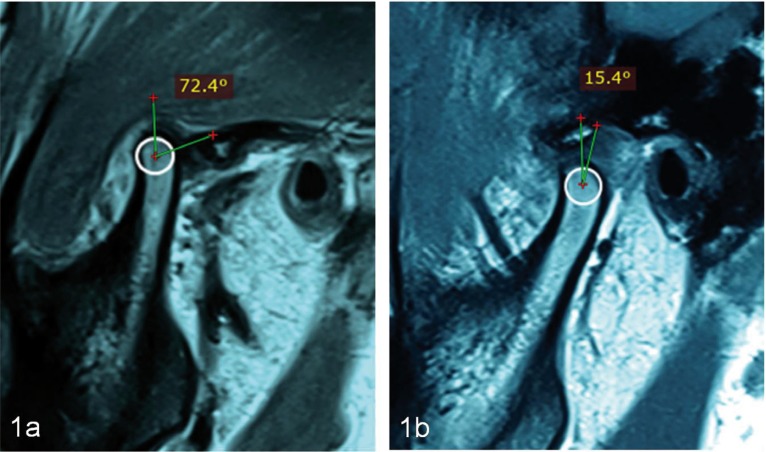
Parameter 1: angle between condylar head and TMJ disk. Pre-operative (a), Post-operative (b).

Angle between the TMJ disk and the condyle head (Parameter 1) measured on pre-operative and post-operative sagittal section of MRI, showed a reduction in angulations in 8 patients. Mean decrease in the angle on right and left TMJ was 30.20±21.581 and 33.10±21.581 respectively, which was statistically significant (*p* value- 0.002 for right TMJ; *p* value- 0.001 left TMJ; Paired t Test) ( Table 3) (Fig. 1a-b).

**Table 3:**
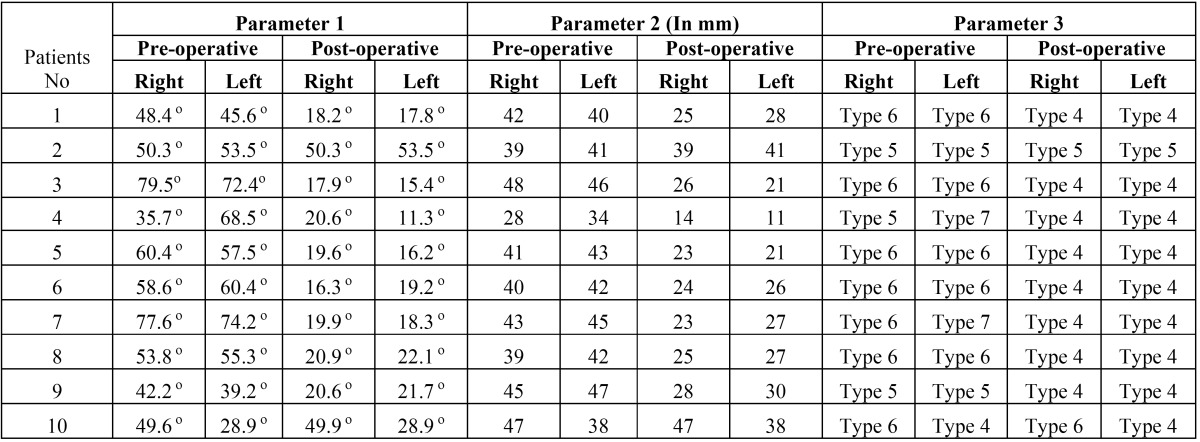
Comparing before and after values parameters of both right and left TMJ.

**Figure 2:**
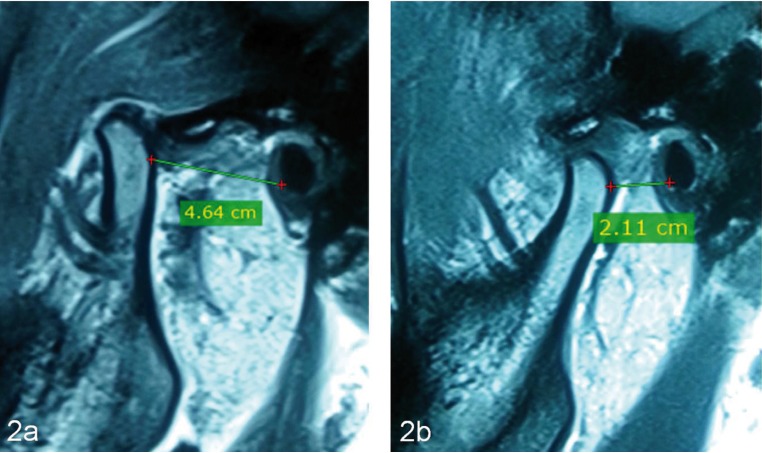
Parameter 2: distance between condyle and EAC. Pre-operative (a), Post-operative (b).

Distance between the condyle and EAC (Parameter 2) measured on pre-operative and post-operative MRI showed decrease in this value in 8 patients. The mean difference of this parameter on pre-operative and post-operative MRI was 13.80±7.671 for right TMJ and 14.80±8.728 for the left TMJ. This difference was statistically significant (*p* value- <.001 bilaterally; Paired t Test) ( Table 3) (Fig. 2a-b).

**Figure 3:**
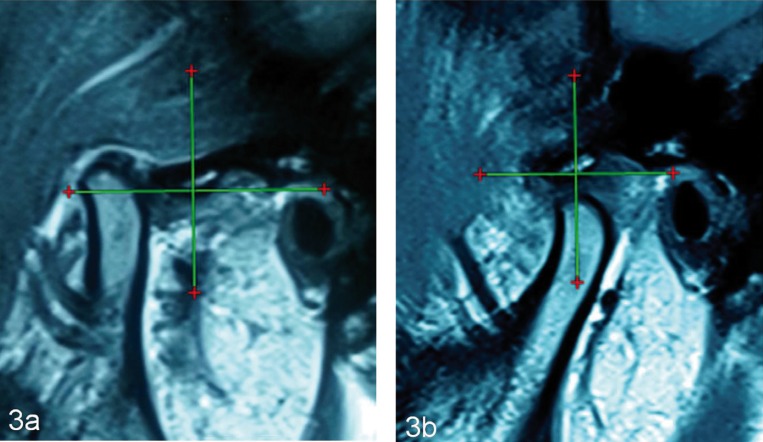
Parameter 3: type of the condyle position. Pre-operative (a), Post-operative (b).

The condyle position at maximum mouth opening was type 6 in 7 patients and type 5 in 3 patients preoperatively on right TMJ. This position changed to type 4 in 8 patients, type 5 in 1 and type 6 in 1 patient at 3 months follow-up. Pre-operative position of left condyle was type 4 in 1 patient, type 5 in 2 patients, type 6 in 5 patients and type 7 in 2 patients. This position changed to type 4 in 9 patients and type 5 in 1 patient 3 months after ABI. The change of condylar position on pre-operative and post-operative MRI was statistically significant (*p* value 0.008 and 0.10 for right and left side respectively; wilcoxon signed ranked test) ( Table 3) (Fig.3a-b)

Postoperative MRI showed no degenerative changes like alteration in joint tissue and cartilage degeneration.

## Discussion

TMJ dislocation is a distressing condition as it causes disability to close the mouth and is often associated with pain and muscle spasm. It results due to an imbalance in the neuromuscular function or structural deficit of the TMJ. Structural defects resulting in TMJ dislocation includes arthritic changes in the condyle (i.e., flattening or narrowing), decrease in the height of the articular eminence, morphological changes of the glenoid fossa, zygomatic arch, and squamotympanic fissure (11,12).

ABI for treatment of chronic recurrent TMJ dislocation was first used by Brachmann in 1964 (9). Subsequently in 1981 Jacobi Hermanns *et al.* reported their experience of treating patients with chronic recurrent TMJ dislocation by ABI with positive outcome (13). Although the mechanism of action of ABI in TMJ is unclear, it is proposed that ABI creates a bed for fibrous tissue formation in the TMJ area, which lead to fibrosis of TMJ. This concept comes from bleeding in the TMJ area after trauma to the condylar region, that leads to formation of fibrous or bony ankylosis (14). During the first few hours or days of ABI, an inflammatory reaction occurs. Release of inflammatory mediators by platelets along with the accumulation of dead and injured cells; lead to oedema of the joint tissue. This results in swelling of the periarticular tissues and the joint becomes physically more difficult to move. Formation of granulation tissue takes place, which is a highly vascularised and loosely organized. Combination of organized blood clots and loose fibrous tissue forms become mature and leads to joint stiffness (15).

In 2011, Candrl *et al.* evaluated the histopathologic effect of ABI in rabbit TMJ (16). The authors did not find any evidences of destructive changes to the osseous or soft tissue components of TMJ. In 2013, Stembirek *et al.* reported the findings of ABI in a pig model. They examined the samples by histopathologic analysis and MRI. Four weeks after the injection they found no rem-nant of blood clots, destructive changes or adhesion inside the TMJ (17).

Hasson and Nahlieli reintroduce ABI technique in clinical practise for treatment of TMJ dislocation in 2001. They injected 5 ml of autologous blood into superior compartment and pericapsular tissue. On follow-up all their cases showed good result with no complications (1). In 2009, Machon *et al.* conducted a similar study in 25 patients. ABI yielded successful outcome in 80% patients. At 1 year follow-up the patients had no episodes of TMJ dislocation and had good functional range of mouth opening (18). Among the studies reported in literature the volume of blood injected in SJC varied from 2 to 5 ml. It is recommended to inject blood into the pericapsular tissue along with SJC for better results (14). In the present study 2 ml of blood was injected into SJC and 1 ml in the pericapsular tissue around the joint. Bayomi *et al.* (2014) (19) and Ahmed *et al.* (2016) (20) reported the success rate of ABI in the range of 70-80%. In the present study single sitting of ABI was administered to 10 patients, 80% of the patients (n=8) had no episodes of recurrence at 3 months follow-up. Two patients who reported with recurrence where advised repeat ABI therapy but refused the same.

In the present study arthrocentesis was combined with ABI to improve the outcome of ABI. Arthrocentesis or lavage and lysis of the TMJ upper compartment markedly improve function and remove pain in many TMJ disorders (21). It forces apart the adherent flexible disc from the fossa, washes away degraded particles and inflammatory components, and decreases the intra-articular pressure. It also helps to reinstate the joint lubrication. The release of disc, elimination of inflammatory products and associated pain allow the rehabilitation of movement, which is the hallmark of joint health (18). The origin of TMJ dislocation is probably related to decreased lubrication which increases friction between the eminence and the disc. The disc, which normally moves together with the condyle, lags behind it, and consequently the condyle slides under and in front of the disc and cannot return to its former position in the fossa. Arthrocentesis of TMJ can restore sliding of the disc, allowing it to move simultaneously with the condyle and preventing the condyle from moving in front of the disc (21). Clicking sound is caused by displacement of the disc; when the mouth is opened, the displaced disc is reduced to the proper relationship with the condyle, causing the clicking noise. Although the effect of arthrocentesis on clicking has not been extensively studied, it is believed that arthrocentesis may reduce the severity of clicking sound and associated pain (21). Arthrocentesis followed by ABI used in the present study resulted in decreased in pain and clicking of TMJ. There was an average reduction of MIO of 9.30 mm (± 6.21). There were no associative complications or facial nerve injury.

MRI is conventionally used to study the soft tissue and osseous components of TMJ, including the disk fossa relationship during functional movement of the joint. Recently MRI has been employed to study the changes in the internal structure of TMJ follo-wing ABI. Candirli C et al. in 2012 evaluated the pathophysiology of ABI using clinical and MRI findings. During the preoperative evaluation, it was identified that the patients had unilateral or bilateral condyles anterior to the articular eminence in the open mouth position, while the postoperative MRI images revealed that the condyles were either at the apex of the articular eminence or posterior to it. Post-operative MRI images showed, no articular cartilage degeneration, disc displacement, or osteoarthritis (22) .Oshiro N *et al.* in 2014 reported MRI findings following ABI for habitual TMJ dislocation. MRI was performed one hour and four and twelve weeks after ABI; three types of findings were described. The first type was similar to hematoma and/or joint effusion in the articular capsule of the TMJ (type I). The second showed sporadic and diffuse T2 emphasis around the TMJ capsule (type II). The third involved a decreased range of condyle movement compared to before ABI (type III) (10).

Result of the present study was evaluated clinically and radiologically (using plain radiographs and MRI). TMJ view taken in maximum open mouth position pre-operatively showed condyle to be anterior to the articular eminence, while the post-operatively radiograph after ABI showed condyle to be at the apex of articluar eminence indicating reduction in the abnormal anterior condylar movement. Quantifiable parameters, including linear and angular measurements were carried on pre and post injection MRI to study and standardise the treatment outcome. Measurement of angle between the TMJ disk and condyle showed a significant reduction postoperatively. This was suggestive of the disk being present over the condylar head during mouth opening after the therapy, which was more posteriorly placed in the pre-operative MRI. Reduction of the excessive anterior translatory movement of condyle was seen after ABI therapy, indicative by the reduction of linear distance between the condyle and EAC.

There was a shift in the condyle position in preoperartive MRI from type 5 and 6 (anterio- inferior and anterio- superior position) to type 4 (inferior position) postoperatively. The above MRI findings of the study showed an improved anatomical and spatial relationship of the osseous and soft tissue components of the TMJ.

ABI is a simple, safe, minimally invasive and cost-effective technique for treatment of chronic recurrent TMJ dislocation. No significant postoperative complication or facial nerve injury was reported in present study. Combination of arthrocentesis with ABI resulted in improvement of pain and clicking sound. MRI evaluation showed reduction of excessive anterior movement of condyle, with an improved anatomical relationship with articular eminence and TMJ disc.

## References

[1] Hasson O, Nahlieli O (2001). Autologous blood injection for treatment of recurrent temporomandibular joint dislocation. Oral Surg Oral Med Oral Pathol Oral Radiol Endod.

[2] Liddell A, Perez DE (2015). Temporomandibular joint dislocation. Oral Maxillofac Surg Clin North Am.

[3] Kuttenberger JJ, Hardt N (2003). Long-term results following miniplate eminoplasty for the treatment of recurrent dislocation and habitual luxation of the temporomandibular joint. Int J Oral Maxillofac Surg.

[4] Akinbami BO (2011). Evaluation of the mechanism and principles of management of temporomandibular joint dislocation. Systematic review of literature and a proposed new classification of temporomandibular joint dislocation. Head Face Med.

[5] Kummoona R (2001). Surgical reconstruction of the temporomandibular joint for chronic subluxation and dislocation. Int J Oral Maxillofac Surg.

[6] Daelen B, Thorwirth V, Koch A (1997). Treatment of recurrent dislocation of the temporomandibular joint with type A botulinum toxin. Int J Oral Maxillofac Surg.

[7] McKelvey L (1950). Sclerosing solution in the treatment of chronic subluxation of the temporomandibular joint. J Oral Surg.

[8] Caminiti MF, Weinberg S (1998). Chronic mandibular dislocation: The role of non surgical and surgical treatment. J Can Dent Assoc.

[9] Brachmann F (1964). Autologous blood injection for recurrent hypermobility of the temporomandibular joint. Dtsch Zahnarztl Z.

[10] Oshiro N, Yoshida H, Uemura M, Suwa F, Morita S (2014). Analysis of MRI findings in minimum invasive treatment for habitual temporomandibular joint dislocation by autologous blood injection around the temporomandibular joint capsule. J Craniomaxillofac Surg.

[11] Güven O (2005). Inappropriate treatments in temporomandibular joint chronic recurrent dislocation: A literature review presenting three particular cases. J Craniofac Surg.

[12] Vasconcelos BC, Porto GG, Neto JP, Vasconcelos CF (2009). Treatment of chronic mandibular dislocations by eminectomy: Follow up of 10 cases and literature review. Med Oral Patol Oral Cir Bucal.

[13] Jacobi-Hermanns E, Tetsch P (1981). Pericapsular autologous blood injection as therapy for habitual temporomandibular joint luxation. Dtsch Zahnarztl Z.

[14] Daif ET (2010). Autologous blood injection as a new treatment modality for chronic recurrent temporomandibular joint dislocation. Oral Surg Oral Med Oral Pathol Oral Radiol Endod.

[15] O'Driscoll SW, Giori NJ (2003). Continuous passive motion (CPM): Theory and principles of clinical application. J Rehabil Res Dev.

[16] Çandrl C, Yüce S, Yldrm S, Sert H (2011). Histopathologic Evaluation of Autologous Blood Injection to the Temporomandibular Joint. J Craniofac Surg.

[17] Stembirek J, Matalova E, Buchtova M, Machon V, Misek I (2013). Investigation of an autologous blood treatment strategy for temporomandibular joint hypermobility in a pig model. Int J Oral Maxillofac Surg.

[18] Machon V, Abramowicz S, Paska J, Dolwick MF (2006). Autologous Blood Injection for the Treatment of Chronic Recurrent Temporomandibular Joint Dislocation. J Oral Maxillofac Surg.

[19] Bayoumi AM, Al-Sebaei MO, Mohamed KM, Al-Yamani AO, Makrami AM (2014). Arthrocentesis followed by intra-articular autologous blood injection for the treatment of recurrent temporomandibular joint dislocation. Int J Oral Maxillofac Surg.

[20] Ahmed S, Ansari M (2016). Treatment of chronic recurrent dislocation of temporomandibular joint by autologus blood injection. Plast Aesthet Res.

[21] Nitzan D (2006). Arthrocentesis- Incentives for using this minimally invasive approach for temporomandibular disorders. Oral Maxillofac Surg Clin North Am.

[22] Candirli C, Yüce S, Cavus UY, Akin K, Cakir B (2012). Autologous blood injection to the temporomandibular joint: magnetic resonance imaging findings. Imaging Sci Dent.

